# Developing a non-invasive diagnostic framework for the fractional flow reserve quantification in left coronary arteries: validation with patient cases

**DOI:** 10.3389/fphys.2026.1875010

**Published:** 2026-07-06

**Authors:** M. Fernandes, F.P. Oliveira, N.D. Ferreira, D. Santos-Ferreira, S. Mushtaq, G. Pontone, R. Ladeiras-Lopes, N. Bettencourt, L.C. Sousa, S. Silva, M.P.L. Parente, S.I.S. Pinto

**Affiliations:** 1 Faculty of Engineering, University of Porto, Rua Dr. Roberto Frias, Porto, Portugal; 2 Institute of Science and Innovation in Mechanical and Industrial Engineering (LAETA-INEGI), Rua Dr. Roberto Frias, Porto, Portugal; 3 Cardiology Department, Unidade Local de Saúde de Gaia e Espinho, R. Conceição Fernandes, Vila Nova de Gaia, Portugal; 4 UnIC@RISE, Department of Surgery and Physiology, Faculty of Medicine, University of Porto, Alameda Prof. Hernâni Monteiro, Porto, Portugal; 5 Department of Perioperative Cardiology and Cardiovascular Imaging, Centro Cardiologico Monzino IRCCS, Milan, Italy; 6 Department of Biomedical, Surgical and Dental Sciences, University of Milan, Milan, Italy; 7 University of Aveiro (UA), Aveiro, Portugal; 8 Institute of Electronics and Informatics Engineering of Aveiro (IEETA), Aveiro, Portugal

**Keywords:** atherosclerosis, computer programming, fractional flow reserve, left coronary artery, non-invasive diagnostics

## Abstract

**Introduction:**

Coronary artery disease (CAD) remains the leading global cause of death. Hemodynamic assessment is typically performed using fractional flow reserve (FFR); however, its invasive nature entails substantial costs and clinical challenges. Non-invasive alternatives are therefore highly desirable. Advances in cardiac imaging, particularly computed tomography (CT), now provide detailed coronary data that can serve as the foundation for computational modeling. This research proposes and validates a tool to numerically predict FFR in patient-specific LCA 3D models segmented from CT scans.

**Methods:**

Using CFD in ANSYS^®^ Fluent, the developed tool employs physiologically informed boundary conditions, a Womersley velocity profile at the inlet and a three-element Windkessel model at the outlets, alongside a simplified Phan–Thien Tanner (sPTT) viscoelastic rheology model for blood. Simulations were conducted under hyperemic conditions to align with how the FFR is currently measured. The non-invasive FFR predictions on 12 patients were compared against both invasive gold standards and commercial HeartFlow^®^ data (Mountain View, CA, USA).

**Results and discussion:**

The numerical results showed a remarkable correlation with invasive measurements (R^2^ = 0.978) and a low average relative error of 3.86% ± 2.01%. Additionally, Bland-Altman analysis indicated high diagnostic precision with a negligible mean bias of −0.015. These metrics suggest improved stability compared to HeartFlow^®^, which showed higher variability (20.84% ± 34.79%) in the cohort of this study. These results are promising; however, given the limited cohort size (12 patients), they should be interpreted as a preliminary proof-of-concept validation rather than a definitive clinical benchmark. The findings of this study suggest that the developed numerical tool has the potential to approximate hyperemic coronary dynamics in a way that is close to the real physiology. Moreover, the developed framework has the potential to be used on-site in medical facilities without costs to assess the functional severity of stenoses and aid the diagnosis of CAD. Larger multi-center clinical trials are required to fully establish the accuracy and generalizability of this tool.

## 1 Introduction

Cardiovascular diseases (CVDs) remain the leading global cause of death, responsible for nearly one in three fatalities annually, with coronary artery disease (CAD) as the most prevalent subtype (Doolub et al., 2024). CAD develops when plaques accumulate inside the coronary arteries, producing stenoses that narrow or occlude the lumen, further leading to myocardial ischemia. Given the fact that coronary blockages can have irreversible consequences without displaying noticeable symptoms, early and accurate detection of CVDs is crucial. Stenoses are evaluated through various anatomical and functional studies. One frequently utilized method for evaluating coronary artery disease is computed tomography (CT), which assists in the initial diagnostic approach and guides decisions regarding medical therapy optimization or the need for further investigations and interventions. If coronary artery disease is identified via CT, findings are typically confirmed by invasive coronary angiography to determine the most appropriate management, ranging from medical therapy to revascularization via stenting or coronary artery bypass surgery (Kamangar et al., 2017; Yang et al., 2023a).

The fractional flow reserve (FFR) is a clinical metric used to quantify the functional significance of coronary artery stenoses. The FFR is considered the gold standard for the diagnosis of CAD. For most authors, ischemia occurs when the FFR of a certain lesion is below 0.80, which means that there is a ratio of 80% between the maximum flow allowed in the presence of a stenosis and the normal maximum flow without the stenosis. To measure this parameter, first the maximization of the flow (hyperemia) is induced by adenosine administration. Then, the pressure distal to the stenosis and the aortic pressure are measured with pressure wires (Fezzi et al., 2025). The FFR is the ratio of these two pressure values, and it is a non-dimensional number varying from 0 (no blood flow) to 1 (unobstructed blood flow).

The measurement of this parameter is performed through an invasive catheterization procedure and, even though it is well-established, this technique is costly, complex, and possibly risky for the patient. A recent study showed that the catheter influences the recorded pressure measurements (Yan et al., 2023). Consequently, there is a need for the development of a non-invasive alternative. The improvement of technology applied in Medicine allows CT-derived 3D models of coronary anatomies to be used with computational fluid dynamics (CFD) to simulate coronary hemodynamics (Edrisnia et al., 2024), opening the possibility of computing FFR without catheterization.

Because the circulatory system operates as a closed loop, local coronary hemodynamics are influenced by all other blood vessels. Since modelling and simulating the full vascular system is computationally impractical, reduced-order representations, such as lumped-parameter (Windkessel) models, or pulsatile velocity profiles (Womersley) are frequently used to improve the accuracy of the CFD results (Antonuccio et al., 2021; Mao and Zhang, 2025; Seresti et al., 2025). Yet, there are authors in the literature that use generic profiles that do not account for the effect of the vascular network or any patient-specific properties (Fossan et al., 2021; Müller et al., 2021; Hashemi et al., 2022).

In addition to its circulation, blood rheology adds another layer of complexity. Newtonian and non-Newtonian shear-thinning rheological models do not capture the viscous and elastic properties of this fluid, which are due to its composition (Zare Jouneghani et al., 2025). Given its complexity, new blood models are still being developed (Spyridakis et al., 2024). With the intent of numerically predicting the FFR, several authors have used blood rheological models that did not account for the viscoelasticity, such as Newtonian models (Csippa et al., 2021; Fossan et al., 2021) and shear-thinning models (Kwon et al., 2014; Chahour et al., 2018). The simplified Phan-Thien/Tanner (sPTT) viscoelastic model, although complex to implement, provides a representation closer to reality, as shown in prior studies (Campo-Deaño et al., 2013; Paz et al., 2025).

One of the remaining gaps identified in the literature concerning non-invasive FFR calculation is the simultaneous use of patient-specific boundary conditions and viscoelastic blood models. Previous CFD studies that assessed the FFR in coronary arteries (Kwon et al., 2014; Chahour et al., 2020; Hoque et al., 2021; Jonášová and Vimmr, 2021) have used Windkessel models in CFD, but did not simulate blood with viscoelastic properties. Hence, the simultaneous implementation of these models has yet to be explored. The novelty of the present study lies in the creation and integration of a fully automated, end-to-end numerical framework that seamlessly couples all these advanced physiological components (3-element Windkessel model, Womersley velocity profile, and sPTT viscoelastic blood rheology) using custom UDFs incorporating patient-specific parameters (heartbeat rate, systole and diastole blood pressures, and local geometry dimensions). The present study simultaneously integrates patient-specific Windkessel boundary conditions for pressure, a Womersley profile for inlet velocity, and blood viscoelasticity through an sPTT model. The goal is to assess the clinical feasibility of this end-to-end pipeline and to calculate the FFR through numerical simulations in a way that is close to the real physiology within a clinically viable timeframe.

In total, 12 patients with varying degrees of stenoses in left coronary arteries (LCAs) are analyzed in this study. Using SimVascular^®^, patient-specific LCAs were segmented from CT images and converted to hyperemic conditions. Afterwards, ANSYS Fluent^®^ was used to perform close to reality patient-specific numerical simulations and obtain a numerical prediction of the FFR. The computed non-invasive FFR results were validated against both invasive measurements obtained from the Cardiology Department of Unidade Local de Saúde de Gaia e Espinho (ULSGE) and predictions provided by HeartFlow^®^. It is a commercial solution that relies on proprietary algorithms with limited transparency and typically requires off-site data processing, potentially raising privacy concerns. For these reasons, a computationally efficient and locally executable, transparent alternative remains a key motivation for this study. Hence, HeartFlow^®^ is included as a comparator for the obtained numerical results, rather than a validation reference, allowing for a performance assessment against an alternative computational approach.

The present study explores whether a locally executable, transparent CFD-based workflow can provide comparable physiological information in a transparent research framework. Moreover, this study aims to create a numerical alternative to the measurement of the FFR through invasive methods, reducing costs and improving patient outcomes, that can be used locally by medical doctors in medical facilities.

## 2 Materials and methods

**Figure 1:**
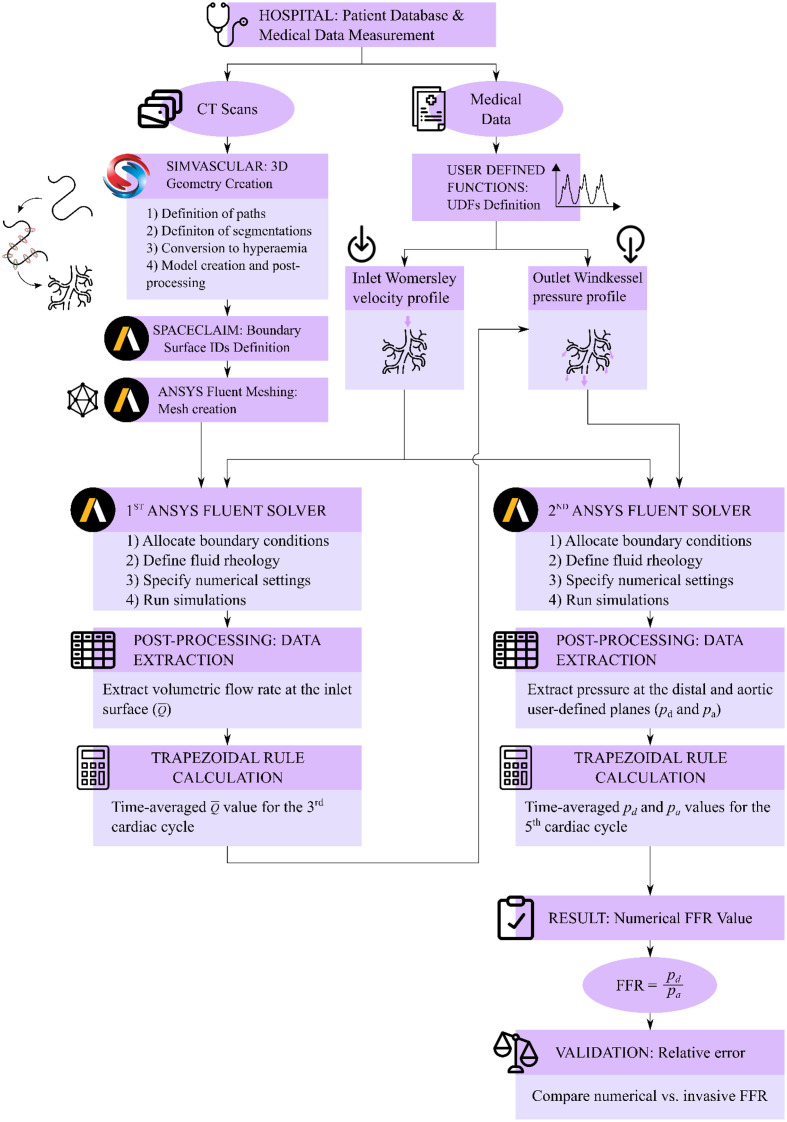
Flowchart used for the numerical prediction of the fractional flow reserve.

The developed workflow is split into 6 different steps: collection of medical data, image segmentation and post-processing of the extracted data to reflect the impact of hyperemia conditions over the anatomy, adaptation of the boundary condition models to the respective patient-specific parameters, numerical simulations, data post-processing (for FFR calculation) and comparison with invasive and HeartFlow^®^ values. This process can be seen in more detail in **Figure 1**. All the different stages are presented in detail in the following subsections.

### 2.1 Patient selection and medical data

In this study, data was collected at the ULSGE from 12 adult patients with several degrees of coronary stenosis (i.e. patients with CCTA-detected left coronary artery stenoses suitable for invasive FFR comparison in vessels with a diameter larger than 2 mm observed in medical imaging) in left coronary arteries. All study cases were sourced via project PTDC/EMD-EMD/0980/2020, supported by FCT-MCTES (Fundação para a Ciência e Tecnologia - Ministério da Ciência, Tecnologia e Ensino Superior), with full informed consent from participants. This was a retrospective validation analysis of prospectively collected/consented patient data from the previously mentioned project. Each entry was processed using a fixed, standardized workflow to ensure independence; no manual tuning or inter-patient modifications were applied. Data anonymization was performed in line with current Portuguese and institutional privacy laws. The study followed the Declaration of Helsinki’s ethical principles and was approved by the ULSGE Ethics Committee on January 27, 2021 (Ref: 53945).

Participants were excluded based on several criteria, including clinical instability (such as recent heart attack, severe heart failure, or arrhythmia) and known allergies to iodine or contrast media. Other reasons for exclusion were reactive airway disease, renal impairment (eGFR< 60 mL/min), and any contraindications to beta-blockers. The authors also excluded individuals who were pregnant or breastfeeding, those with implantable cardiac devices, patients with a left ventricular ejection fraction under 50%, and anyone with a history of coronary interventions like PCI or CABG. The final sample contained 12 patients. It is important to highlight that the final patient cohort is quite small. As such, the results constitute a preliminary proof-of-concept and are insufficient to support a definitive clinical benchmark.

All patients underwent coronary computed tomography angiography (CCTA) for anatomical assessment and invasive FFR measurements through catheterization. All CCTA was performed using a Siemens Healthineers Somatom Force, a third-generation dual-source system, following a standardized protocol to maintain cross-dataset uniformity. To stabilize heartbeat rates (HBR), patients received oral metoprolol or intravenous esmolol. Sublingual nitrates were administered to minimize vasomotor tone and promote the most optimal coronary vasodilation. Calcium scoring utilized ECG-triggered high-pitch scans at 120 kV tube voltage. For the CCTA itself, the acquisition mode was determined by the heartbeat rate: patients with an HBR under 65 bpm underwent prospectively ECG-triggered high-pitch spiral scans, while those with HBR equal or above 65 bpm were imaged via standard prospectively ECG-triggered sequential with padding acquisition.

For both cases, contrast delivery involved a 50–70 mL bolus of Iopromide (370 mg/mL; Bayer Schering Pharma, Berlin, Germany) followed by a 30 mL saline solution injection, both at rates of 4.5–6.0 mL/s using a dual-head power injector (Stellant, Medrad, Indianola, USA).

Radiation exposure was managed through CARE kV (120 kV; 300 mAs/rotation, Siemens Healthineers, Germany). The collimation and rotation time, parameters of CCTA, were 192×0.6 mm, and 0.25s, respectively. Finally, axial reconstructions of the coronaries were generated using the SAFIRE iterative algorithm (kernels I26 or Br40, strength 3, Siemens) with slice thicknesses of 0.6 mm and increments of 0.3 mm.

Resting-state hemodynamic parameters such as systolic and diastolic blood pressure (SBP and DBP, respectively) as well as HBR were collected for each patient. The invasive FFR was obtained using pressure sensors introduced into the coronary arteries under pharmacologically-induced hyperemia. The FFR value for each patient was defined as the ratio of mean distal coronary pressure (p_d_), measured approximately 20 mm downstream of the stenosis, to the mean aortic pressure (p_a_) at the coronary ostium during maximal hyperemia (Equation 1).

(1) $$FFR=\frac{p_{d}}{p_{a}}$$

Maximum hyperemia is achieved when the vessel is dilated through the intravenous infusion of adenosine. A quantity of 140 µg/kg/min is the standard dose for pharmacological stress testing to induce maximal coronary vasodilation, used in clinical practice (Christensen et al., 2025). This vasodilation leads to changes in heartbeat rate (increases 24 bpm), blood pressure (decreases 6 mmHg), flow resistance (decreases by a factor of 4.17), as well as an increase of the cross-sectional area of the vessel by a factor of 2.04 (Sharma et al., 2004).

HeartFlow^®^ FFR values were independently computed by the HeartFlow^®^ platform after hospital submission of the same CCTA datasets, without any local post-processing or modification.

The summary of the medical patient data table is provided in **Table 1**, while the detailed version is presented in **Table 2**.

**Table 1:** Data of the patients included in the study.

Number of patients	12
Height (cm)	164.7 ± 8.3
Weight (kg)	72.2 ± 11.6
Invasive FFR values	0.694 ± 0.181

**Table 2:** Detailed data of the patients included in the study.

Patient number	Blood Pressure (mmHg)	Heartbeat Rate (bpm)	Height (cm)	Weight (kg)	Invasive FFR values
1	136/67	59	155	73	0.58
2	149/91	56	175	79	0.81
3	132/72	54	162	59	0.61
4	128/76	68	170	64	0.6
5	152/87	48	154	54	0.81
6	128/69	52	169	83	0.9
7	125/65	60	162	71	0.5
8	138/95	56	174	72	0.29
9	138/93	68	175	80	0.79
10	106/57	50	160	73	0.9
11	137/76	45	150	60	0.77
12	124/78	60	170	98	0.77

### 2.2 3D model generation

The left coronary artery (LCA) trees contain several vessels: the left main coronary artery (LMCA), left anterior descending artery (LAD), with the subbranches Proximal LAD, Medial LAD, Apical LAD, first (D1) and second diagonal branch (D2), circumflex artery (CX), with the subbranches Proximal CX, obtuse marginal branch (OM), distal CX, left posterolateral branch (PL) and, if left-dominant, posterior descending artery (PDA). All these vessels must be accounted for in the numerical simulations to improve the accuracy of the results.

The construction of the three-dimensional patient-specific LCA anatomy was performed using the open-source software SimVascular (Updegrove et al., 2017). Several works of the literature have applied this software to accurately segment coronary arteries for numerical simulations that produced satisfactory results (Alzhanov et al., 2023; Yang et al., 2023b). CT images, captured at the ULSGE, were uploaded to the program and segmented manually to isolate the left coronary artery tree. The number of CT slices available per patient was, on average, 330 ± 76.

**Figure 2:**
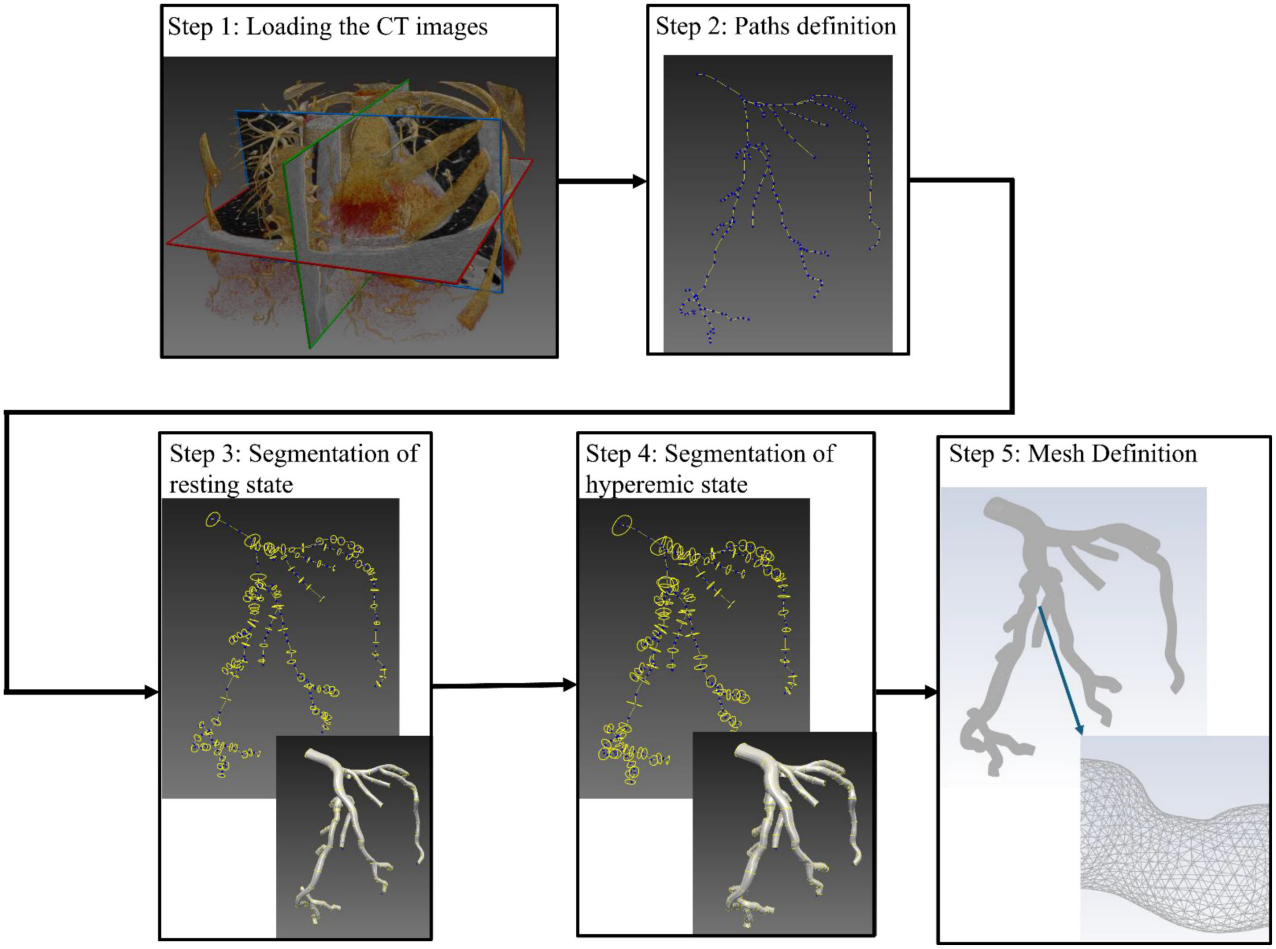
Image segmentation procedure of obtaining a mesh suitable for numerical simulations from CT images.

The complete segmentation procedure on SimVascular follows a general structure containing three steps: the definition of the paths, the definition of the segmentations and the creation of the model. The different stages used in the segmentation process and mesh creation are detailed in the following subsections, and shown in **Figure 2**, which were repeated for every patient.

#### 2.2.1 Definition of paths

The paths were defined as the centerlines of each vessel and are a series of points chosen over the images. The points are automatically connected by the software to form a curvilinear spline that best fits the provided data. The choice of the number and location of the points was defined by the user on a per-patient basis, but the density of points was higher at the stenoses and in zones with higher tortuosity. The points were chosen in the axial view and, to ensure their centrality over the cross-section of the artery, all points were assessed in perpendicular views (sagittal and coronal). These points were also assessed by viewing the 3D view created automatically by the software, to ensure that no vessels were missing and that the points were correctly placed inside each vessel. The accuracy of the defined points was visually assessed for all paths.

Overall, the average number of paths per patient was 14 ± 4, and the average patient also had 14 ± 4 points per path.

On a path basis, the software can automatically define the direction of the vector tangent to the centerline at each point. In addition, it is also able to automatically create new points that belong to the centerline. This is crucial since the software can, then, create cross-sectional planes, which are perpendicular to the centerline, at each point. These planes are used to draw the shape of the cross-section.

#### 2.2.2 Definition of segmentations and conversion to hyperemia

Following centerline definition, the next step is drawing the boundaries of the lumen, which are heightened by the contrast administered during the image capturing procedure. The authors found that the manual process led to more accurate shapes as opposed to the machine learning process offered. The method used was the spline tool, which created a closed curvilinear polygon from points located at the boundary. The software is, then, able to loft together all the 2D shapes into a 3D representation of the vessel lumen, which is useful to assess the anatomy of the generated geometries in comparison to the real anatomy.

Afterwards, the cross-sectional area was increased by a factor of 2.04, to replicate hyperemia conditions (Sharma et al., 2004), leading to the geometry reflecting the maximum vasodilation state. This process was performed automatically using a Python script to process the cross-sectional lumen masks previously delineated for each vessel. The shape of the boundaries and the overall anatomy remained the same, but some errors arose between main and side vessels. Since the scale factor is systematically applied to all cross sections, the expansion of larger vessels, such as the LAD or LCX, resulted in intersections with the closer branches. Therefore, manual adjustments were made to ensure that smaller branches remained at a distance from the main vessels. This procedure will lead to a geometry with a different spatial distribution than the original anatomy, but, considering that the cross sections and connections are kept, no meaningful changes to the simulated flow were expected.

#### 2.2.3 Creation of the model

The generated segmentations are selected to form the model of the left coronary tree. The surface of the model created in SimVascular was subjected to a manual local smoothing operation with more focus on bifurcations and trifurcations to smooth the transitions between vessels, increase the closeness between 3D geometries and the real arteries, and also make sure that the resulting model would be suitable for computational fluid dynamics (CFD) simulations.

Readers can find more information regarding this software and its sequential process in (Updegrove et al., 2017). However, the duration of the process increases with the complexity of the vessel networks. In fact, the segmentation of left coronary arteries is more time-consuming than, for instance, the segmentation of right coronary arteries (RCAs) since the former contains more branches and vessels than the latter. In fact, the RCA usually possesses 4 branches: the conus artery, sinoatrial (SA) nodal artery, right marginal artery, and the posterior descending artery (PDA). In addition, the RCAs usually have less paths and less anatomy variation (Sake et al., 2020).

To accurately define the surfaces for boundary condition implementation across all left coronary artery (LCA) models, ANSYS SpaceClaim^®^ was used. Surface identification allows the boundary conditions to be applied to the correct cross-sectional area. This program was also used to align the inlet surface with the y0z plane (*x=0*), and the centroid of this surface was aligned to the origin point (with coordinates *x=y=z=0*). This aspect is relevant for boundary condition definition, performed in Section 2.4.

### 2.3 Mesh creation

Classical mesh convergence would require iterative mesh refinement for every single patient case. Given the number of patients and the clinical feasibility objective of this tool, performing a complete mesh convergence study for each patient geometry would substantially increase processing times and hinder the on-site applicability of the tool. Instead, the authors opted to employ fault-tolerant meshing (FTM) in ANSYS Fluent^®^, which allows creating a suitable mesh, and, therefore, stable numerical simulations, by creating a closed surface over possibly existing irregularities in the models.

All the meshes contain tetrahedral elements with element sizes roughly between 0.25mm and 0.55mm and with refinement in the sections with smaller cross-sectional area, like the stenoses. Additionally, to ensure the integrity of the mesh, the maximum skewness parameter was defined as 0.7. Skewness is a parameter commonly used for the evaluation of the quality of the mesh and represents how close each tetrahedron of the mesh resembles the ideal equilateral element (with all internal angles equal to 60 degrees). To ensure solution convergence and prevent numerical errors, it is critical that the maximum skewness value remains below 0.95 (Miranda et al., 2021), and the authors verified that the skewness of all patients did not exceed 0.7. No boundary layers were used in the mesh.

Based on prior experience of the research group and conclusions drawn from previous studies, mesh convergence in coronary artery CFD simulations was consistently observed to be reached at approximately 300000 elements for the key hemodynamic quantities relevant to FFR estimation, namely pressure fields (Pinto et al., 2020). For the present study, the created meshes possessed around 900000 elements without applying any refinement. However, since the goal of this study is to calculate the FFR, the mesh around regions with lower cross-sectional area values, such as stenoses, were further refined. This process resulted in a rise of the number of total tetrahedral elements to close to 1000000 for all patients. Hence, the authors ensure that the chosen meshes for all patients are adequately refined for numerical simulation and further FFR calculation.

### 2.4 Boundary conditions definition as user-defined functions

Velocity and pressure boundary conditions should be able to approximate blood flow settings during hyperemia, the conditions in which the FFR is measured. A Womersley pulsatile velocity profile was imposed at the inlet of the vessel, while the outflow at the outlets was modelled with a 3-element Windkessel lumped parameter model, accounting for proximal and distal resistance as well as arterial compliance. It is important to highlight the patient-specific nature of the boundary condition mathematical models, which were adapted according to the number of outlets, the area of the inlet and outlet surfaces, and the medical information of each patient, such as heartbeat rate, systolic and diastolic blood pressures. These boundary conditions are directly governed by individual patient data via User-Defined Functions (UDFs). Specifically, the 3-element Windkessel model calculates parameters for each outlet by explicitly incorporating that patient-specific measured systolic and diastolic blood pressure, their heartbeat rate during hyperemia, and the individual anatomical outlet areas of the reconstructed coronary branches. Similarly, the inlet flow dynamics are modelled using a Womersley profile that is directly scaled by the patient-specific inlet cross-sectional area and their hyperemic heartbeat rate. Any remaining parameters that could not be measured on a case-by-case basis are literature-derived.

For the inlet surface, to capture the pulsatile and cyclic nature of the circulatory system, researchers frequently use a Womersley model to characterize the inlet flow and assess the influence of transient inertia and viscous forces on the flow (Boileau et al., 2018; Yin et al., 2019; Udupa et al., 2025). This corresponds to the blood leaving the aorta, coming from the left ventricle. Since this is commonly used in the literature to model blood flow entering the coronary artery, the scaled generic waveforms used in the present study are consistent with physiologically representative LCA inlet blood flow patterns reported in the literature. Therefore, the authors consider the patient-specific Womersley model as an appropriate inlet boundary condition for coronary CFD simulations.

The fluid velocity, *u*, is modelled as a multi-variable function of radius, *r*, longitudinal position along the centerline, *x*, and time, *t* (Equation 2):

(2) $$u\left(r,x,t\right)=f\left(r\right)\bar{u}\left(x,t\right)$$

where $\bar{u}$ represents the axial-average velocity, defined as (Equation 3):

(3) $$\bar{u}=\frac{2}{R^{2}}\int_{0}^{R}u\left(r,x,t\right)r\;dr$$

The variable *r* varies from 0, at the centerline, to the radius *R*, at the wall. The simulations considered the 3D geometry as having rigid walls, which means the radius is not a function of time. As previously mentioned, the inlet surface was placed at *x=0* plane and its centroid at the origin point. Hence, *r* is calculated as (Equation 4):

(4) $$r=\sqrt{y^{2}+z^{2}}$$

This radius value was normalized according to the inlet radius of each patient. The profile function *f(r)* is determined by the flow regime which, in this case, is a Womersley model applied to an incompressible fluid (Madhavan and Kemmerling, 2018), equal to (Equations 5–7):

(5) $$f\left(r\right)=R\left\{\frac{\Lambda J_{0}\left(\Lambda \right)-\Lambda J_{0}\left(\frac{r}{R}\Lambda \right)}{\Lambda J_{0}\left(\Lambda \right)-2J_{1}\left(\Lambda \right)}\right\}$$

(6) $$\Lambda =\left(\frac{i-1}{\sqrt{2}}\right)\alpha$$

(7) $$\alpha =\sqrt{\frac{\omega \rho }{\mu }}R$$

In the previous expressions, ℜ denotes the real part of the complex function, *J_0_* and *J_1_* are the Bessel functions of the null and first order respectively, *i* is the imaginary unit, and *α* is the Womersley number (representing the ratio of transient inertial to viscous forces). The variables *ρ* and *ω* represent the fluid density and angular frequency, respectively, while *R* is the arterial radius (San and Staples, 2012). The angular frequency is derived from the patient-specific cardiac period, *T*, which depends on the heartbeat rate (HBR) (Equations 8, 9):

(8) $$T=\frac{60}{HBR}$$

(9) $$\omega =\frac{2\pi }{T}$$

It is important to highlight how, in maximum vasodilation settings, there is more blood circulating per unit of time (since the cross-sectional area of the arteries is larger), and such can only occur if the heart is pumping faster. The HBR increase by 24 bpm in hyperemic conditions (Sharma et al., 2012), so this change was applied. To implement the Womersley velocity profile numerically, flow waveforms are reconstructed from a literature validated velocity inlet waveform using a Fourier series (Miranda et al., 2021). This involves fitting harmonic coefficients *a_i_* and *b_i_* to the velocity inlet data (present in **Table 3**).

**Table 3:** Harmonic coefficients used in the Fourier-derived velocity profile used in the inlet boundary of all patients.

*i*	0	1	2	3	4	5
*a_i_*	26.16	5.282	-0.909	-2.733	2.509	-0.268
*b_i_*	0	-4.866	-1.040	0.2598	2.111	-1.372

Moreover, to replicate maximum vasodilation, the inlet velocity is adjusted by a factor of 2.16 (Sharma et al., 2012):

(10) $$u\left(r,t\right)=2.16\frac{AR^{2}}{\pi i\mu \alpha^{2}}\left(1-\frac{J_{0}\left(\alpha \frac{r}{R}i^{\frac{3}{2}}\right)}{J_{0}\left(\alpha \;i^{\frac{3}{2}}\right)}\right)e^{i\omega t}\;$$

In the previous Equation 10, *A* is the amplitude of the pressure gradient. When it comes to the outlet surfaces, in this study, a three-element Windkessel model was implemented. This model is prevalent in numerical recreation of blood flow in established literature and has the ability to provide accurate physiological results (Madhavan and Kemmerling, 2018; Deyranlou et al., 2020; Jonášová and Vimmr, 2021).

Regarding pressure, a three-element Windkessel model was used to model outlet pressure. There is a strong physiological correlation between a vessel structural dimension (outlet area) and the myocardial mass it perfuses, which means that downstream microvascular resistance and flow vary for different patients, and generic profiles should be avoided (Westerhof et al., 2009). Therefore, some parameters of this model were calculated using patient-specific data, to better mimic blood flow of each patient.

This boundary condition represents the hemodynamics of the peripheral arterioles and capillaries through three components: proximal resistance *R_p_*, distal resistance, *R_d_*, and total arterial compliance, *C_a_*. *R_p_* represents the characteristic impedance and resistance of the coronary arteries immediately downstream from the 3D geometry domain. *R_d_* accounts for the high-resistance microvascular bed of smaller vessels like arterioles and capillaries. *C_a_* represent the elasticity of the downstream vessel walls (Westerhof et al., 2009). The resistance values are proportional to the area of the outlets, the average pressure and flow rate at the inlet (Equations 11, 12):

(11) $$R_{p},R_{d}\propto \frac{A_{i}\times \frac{\bar{p}}{\bar{Q}}}{\sum A_{i}}$$

(12) $$\bar{p}=\frac{SBP+2\;DBP}{3}$$

The average inlet pressure, $\bar{p}$, is defined in Medicine as a weighted average of the systolic and diastolic blood pressure values (*SBP* and *DBP*, respectively) whose weights are proportional to the duration of each phase within each cardiac cycle. $\bar{Q}$ is obtained in the first set of numerical simulations, where the Windkessel model is applied, but the outlet pressure is null and averaged over the cardiac cycle through a trapezoidal rule.

The compliance parameter for each outlet is also different for each coronary branch using an area-proportional scaling approach, following the physiological formulations established for arterial trees (Deyranlou et al., 2020). The individual outlet compliance (C_i_) is calculated by multiplying the total estimated coronary compliance (C_total_) by the ratio of that individual outlet cross-sectional area (A_i_) to the sum of all outlet areas across the modelled LCA network (Equation 13):

(13) $$C_{i}=C_{total}\frac{A_{i}}{\sum A_{i}}$$

This approach is rooted in vascular morphometry principles, which dictate that larger downstream vascular beds possess a higher capacity to expand and store blood volume during the cardiac cycle. The total coronary compliance (C_total_) is dynamically tuned using patient-specific global physiological markers, specifically the hyperemic heartbeat rate and systolic and diastolic blood pressure, to ensure that the resulting pressure waveforms are close to the real physiology (Deyranlou et al., 2020). The resistance values are assigned using a similar area-proportional approach, as described previously.

Using this configuration, the proximal resistance is connected to each arterial outlet and is placed in series with the distal resistance. The compliance element is positioned in parallel with the distal resistance. For these models, the external and the right atrium pressure are assumed to be zero (Jonášová and Vimmr, 2021). The model is described by the following differential equations (Equations 14–16):

(14) $$\frac{dp_{d}}{dt}+\frac{p_{d}}{C_{a}R_{d}}=\frac{Q_{0}}{C_{a}}\;$$

(15) $$p_{0}=p_{d}+R_{p}Q_{0}$$

(16) $$\left(1+\frac{R_{p}}{R_{d}}\right)\frac{Q\left(t\right)}{C_{a}}+R_{p}\frac{Q\left(t\right)}{dt}=\frac{dp\left(t\right)}{dt}+\frac{p\left(t\right)}{C_{a}R_{d}}$$

In the previous, *Q_0_* and *p_0_* represent the initial flow rate and pressure at the outlet, respectively, while *Q(t)* and *p(t)* describe the temporal evolution of these parameters throughout the cardiac cycle. The pressure was decreased by 6mmHg to replicate hyperemic flow (Sharma et al., 2012).

### 2.5 Blood rheology characterization

In this study, blood was defined as a non-Newtonian viscoelastic fluid. This is because of its composition since it is a dense suspension of cellular elements, primarily red blood cells (RBCs), which possess the ability to aggregate into ‘rouleaux’ structures at low shear rates and deform under high shear stress. This dynamic behavior results in shear-dependent viscosity and elastic energy storage, which cannot be described through Newtonian and shear-thinning models (Zare Jouneghani et al., 2025).

The Simplified Phan-Thien/Tanner (sPTT) constitutive model was selected because it effectively captures critical viscoelastic behaviors like the adaptation to variation in the vessel geometry (like the presence of stenosis and bifurcations), the normal stress differences caused by the elongation and relaxation of RBCs during the cardiac cycle (Sharma et al., 2012; Campo-Deaño et al., 2013).

The choice of the sPTT model over Newtonian or shear-thinning alternatives is supported by a prior study from our research group, in which a direct comparison was performed between Newtonian, shear-thinning (Carreau, Carreau-Yasuda, Casson), and viscoelastic models. That study explicitly demonstrated that the sPTT model is critical for better capturing energy losses and flow profiles necessary for accurate FFR predictions in coronary arteries, where simpler models failed to adequately reproduce the pressure fields (Fernandes et al., 2024).

The sPTT model is described as (Equations 17–20):

(17) $$\tau =\tau_{s}+\tau_{e}$$

(18) $$\tau_{s}=\mu_{s}\dot{\gamma }$$

(19) $$\left[1+\frac{\epsilon_{k}\lambda_{k}}{\mu_{e,k}}tr\left(\tau_{e}\right)\right]\tau_{e}+\lambda_{k}{\overset{▽}{\tau }}_{e}=\dot{\gamma }\mu_{e,k}$$

(20) $${\overset{▽}{\tau }}_{e}=\frac{D\tau }{Dt}-{\left(\nabla \cdot u\right)}^{T}\tau_{e}-\tau_{e}\left(\nabla \cdot u\right)$$

In this model, the shear stress of blood, ***τ***, is the sum of a solvent part, ***τ****_s_*, and an elastic one, ***τ****_e_*, which are proportional to their respective viscosities, *μ_s_* and *μ_e_*, and the shear rate vector, $\dot{\gamma }$. The vector ***u*** is the velocity vector of the flow field and the extensibility coefficient is represented by *ε*. Additionally, the operator *tr* indicates the trace, and ∇ is the upper-convected time derivative. This model has four distinct modes, and each is defined by *k*. The values of *µ_e,k_*, *λ_k_*, and *ϵ_k_* were defined by (Campo-Deaño et al., 2013), and are displayed in **Table 4**.

**Table 4:** Parameters of the four-mode simplified Phan-Thien/Tanner (sPTT) model used to reproduce the viscoelasticity of blood (Campo-Deaño et al., 2013).

Mode, k	1	2	3	4	5 (solvent)
*µ_e,k_* (Pa×s)	0.05	0.001	0.001	0.0016	0.0012
*λ_k_* (s)	7	0.4	0.4	0.006	0
*ϵ_k_*	0.2	0.5	0.5	0.5	0

### 2.6 Numerical techniques definition and data extraction

Hemodynamic simulations were performed for all the patient-specific left coronary arteries using the ANSYS Fluent^®^ solver. Simulations were conducted on a high-performance workstation (Intel Core i9 Extreme, 64 GB RAM). The SIMPLE algorithm was used and, to ensure numerical precision, second-order spatial and temporal discretization schemes were employed. The density of blood was defined as a constant (incompressible fluid) and equal to 1060 kg m^-3^. All simulations assumed rigid arterial walls, since previous literature showed that fluid-structure interaction (FSI) has a negligible impact on hemodynamics (Miranda et al., 2021).

The boundary conditions are continuous mathematical models that, prior to implementation in ANSYS Fluent^®^, must be converted into a system of discrete algebraic equations to be solved numerically. To minimize numerical diffusion throughout the computational domain and ensure high accuracy, second-order spatial and temporal discretization schemes were selected in the Fluent solver settings when implementing the UDF-defined boundary conditions. The sPTT model was also implemented as UDFs, and, to account for the four modes, 24 scalars were defined. The latter were coupled with the momentum equations using a second-order upwind scheme.

Each patient case required two separate numerical simulations in different settings because of the volumetric flow rate $\bar{Q}$. The only difference between the two numerical simulations were that the outlet pressure was null in the former and used the 3-element Windkessel model in the latter. Looking at the waveforms produced in both simulations, the authors noted that the former one converged on the third cardiac cycle, while the latter only converged on the fifth, so the last cardiac cycle values were used for the calculation of $\bar{Q}$, and the pressure values (*p_d_* and *p_a_*), respectively. Numerical convergence was assessed by a 10–^6^ residual threshold. The numerical simulations performed in this study were transient. A time-step of 0.005s was used, with a maximum of 20 iterations per time-step. This time-step size and maximum number of iterations per time step have been used in previous studies from our research group and provides excellent numerical precision (Pinho et al., 2019; Pinto et al., 2020; Miranda et al., 2021). The 20 iterations per time-step represents the maximum allowed number of iterations within the solver settings; in practice, stable convergence was consistently observed within this limit based on our previous experience. To ensure the diagnostic results are free from initialization bias the FFR values were calculated by taking the temporal average over the fully stable and periodic 5th cardiac cycle. It is known that three-dimensional cardiovascular simulations typically require several cardiac cycles before reaching a periodic solution (Pfaller et al., 2021), so the authors have also used this approach in previous works (Pinho et al., 2019; Pinto et al., 2020; Miranda et al., 2021).

The aortic and distal planes, used to obtain the pressure values used in the calculation of the FFR, were manually defined on ANSYS Fluent^®^. The *p_a_* pressure was measured at an aortic plane placed 10μm after the inlet. This distance is small enough to capture the true aortic pressure while remaining numerically isolated from boundary effects. On the other hand, *p_d_* was measured at a plane 20 mm downstream of the stenosis. The plane was defined by three coordinates, which were first identified in ANSYS SpaceClaim^®^. Here, by analysis of the location of the stenoses, signaled by medical doctors in the CT images of each patient, the plane perpendicular to the centerline 20 mm downstream of the stenosis center was picked. This process was manual and complex due to the natural complexity of left coronary arteries. These plane positions were defined to mirror the clinical placement of physical FFR pressure wires.

Both pressure values were integrated over the cardiac cycle using the trapezoidal rule to ensure the results reflected the hemodynamic significance of the stenoses under patient-specific flow conditions.

### 2.7 Statistical analysis

All statistical analyses were performed in Microsoft Excel. The relative error between numerical and invasive FFR values was calculated as Equation 21:

(21) $$\text{Relative error }(\%)=\frac{{\text{FFR}}_{\text{numerical}}-{\text{FFR}}_{\text{invasive}}}{{\text{FFR}}_{\text{invasive}}}\times 100$$

, and equivalently between HeartFlow^®^ and invasive FFR values. The Bland-Altman analysis was performed following the method of Bland and Altman (1986), computing the mean bias as the mean of the differences between numerical and invasive FFR (as well as between HeartFlow^®^ and invasive FFR separately) and the 95% limits of agreement as mean bias ± 1.96 × standard deviation (SD) of the differences. The 95% confidence intervals were derived using the t-distribution for n = 12. More information regarding these equations can be found at (Bland and Altman, 1986).

The R² coefficient was computed as the squared Pearson correlation coefficient between numerical and invasive FFR values. All analyses were conducted on the same 12 patients. All reported standard deviations are sample SDs. The analyses were not pre-registered, as this is an exploratory proof-of-concept validation study.

## 3 Results and discussion

This section contains the results obtained from the numerical simulations, as well as their discussion. In particular, and for all the analyzed patients, velocity and pressure three-dimensional distributions were plotted in ANSYS^®^ CFD-Post, at systolic peak. The systolic peak corresponds to the instant of maximum aortic pressure, maximum translesional pressure gradient, making it a hemodynamically representative moment for the assessment of stenosis severity and coronary flow reserve.

Pressure contours are presented first, as they allow direct identification of the most functionally impactful stenoses through the pressure gradients they generate. Velocity contours are presented in the following subsection to quantify how those velocity-driven flow disturbances translate into pressure losses along the coronary tree, which is necessary for FFR computation.

This allows the understanding of how the coronary flow at these critical time instances is, and in particular areas, such as bifurcations and the stenoses.

In addition to these plots, the numerical FFR values obtained from the numerical simulations are compared to the invasive hospital FFR values as well as the HeartFlow^®^ predicted FFR values.

In each of the following subsections, quantitative results are presented first, followed by their appropriate interpretation and discussion.

### 3.1 Pressure distribution assessment

**Figure 3:**
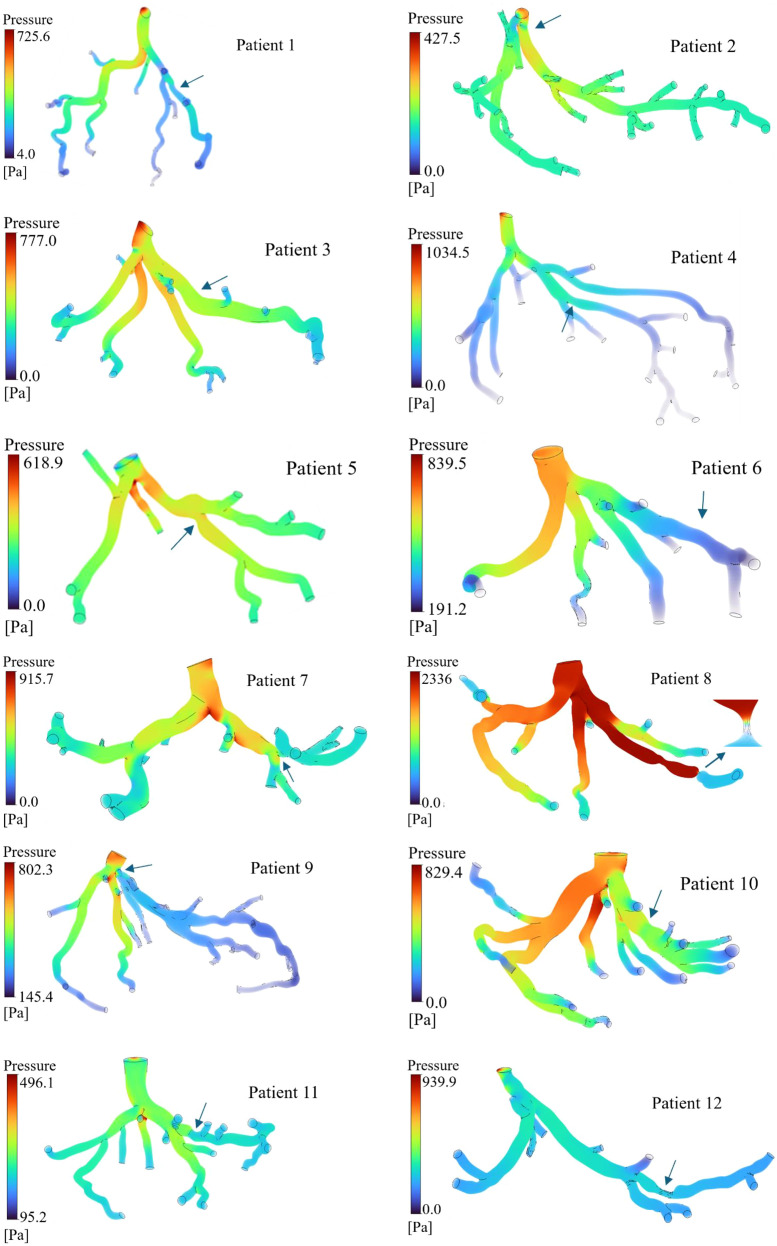
3D pressure distribution at the systolic peak for all patients. The stenoses are highlighted with arrows.

**Figure 3** displays the 3D pressure distribution maps for the entire patient cohort, captured during the systolic peak. The locations of the stenoses are specifically indicated by arrows, allowing direct assessment of the pressure gradients induced by each stenosis.

The pressure near the aorta is consistently higher than downstream in the systole, which is to be expected in left coronary artery trees. Systolic inlet pressures across the cohort, given by the maximum pressure value achieved, vary from approximately 430 Pa to 1450 Pa in most patients. This pressure range reflects the elevated driving force of ventricular contraction and the increased flow that occurs during maximal vasodilation. Patient 8, the one in the patient cohort with the most severe stenosis (invasive FFR of 0.29), exhibited the highest inlet pressure of 2336 Pa.

Substantial pressure drops are evident across the stenotic regions, since they have smaller cross-sectional areas. The images allow the location of the most physiologically impactful lesions within each coronary tree. The most substantial gradients appearing in patients 1, 7, and 8, which correspond to the patients with the lowest invasive FFR values. The strong correlation between the visual pressure gradients in the previous figure and the low invasive FFR values suggests that these pressure contours are highly effective for the non-invasive prediction of functional lesion severity, as well as the visual assessment of its impact on blood flow.

The stenoses lead to greater fluid resistance when flowing through them, which leads to loss of energy. This is caused by viscous friction in the course of the stenosis and separation losses at the stenosis exit. As a result, for all patients, the pressure increases after the stenoses but never recovers to the initial value. Hence, stenoses permanently affect coronary flow. This is explained by the Venturi effect and Bernoulli’s Principle. This is most evident in patients 1, 5, 9, and 11. In the remaining cases, the recovery of pressure requires a longer distance downstream of the stenoses, which was not captured during the segmentation stage due to small vessel diameter. The impact caused by the stenosis is irreversible and leads to lower perfusion pressure to the myocardium (the heart muscles), reducing its pumping ability.

Overall, the findings presented in this section seem to align with physiological blood flow dynamics observed in the literature (Nichols et al., 2011; Gao et al., 2020). The pressure fields qualitatively reproduce well-established hydrodynamic patterns, with separation effects becoming increasingly pronounced in the more severe stenoses of this cohort. In addition, the results are consistent with the expectation of longer recirculation zones and higher pressure losses as stenosis severity increases.

### 3.2 Velocity distribution assessment

**Figure 4:**
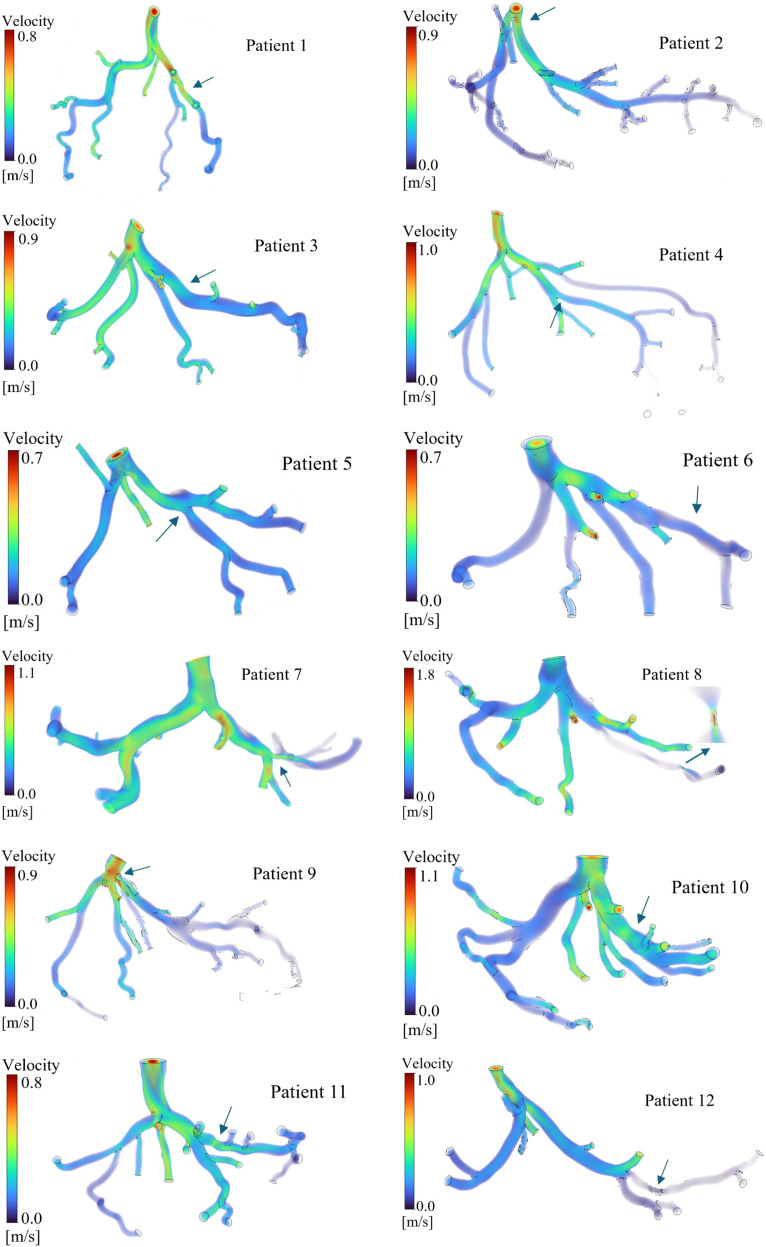
3D velocity distribution at the systolic peak for all patients. The stenoses are highlighted with arrows.

**Figure 4** illustrates the 3D velocity distribution maps for all patients during systolic peak. Similar to pressure, these maps highlight the flow dynamics within the vessels, with arrows marking the stenotic regions to demonstrate how they impact blood flow velocity.

In all patient cases, the presence of stenoses led to, as seen in the previous subsection, a sharp decrease in pressure. Here, it is possible to see how the velocity increases as blood flows through the stenosis and loses speed as it leaves the narrowing, after the cross-sectional area increases. During systole, peak velocities at stenotic throats range from approximately 0.7 m/s in milder cases (patients 5 and 6, with invasive FFR of 0.81 and 0.90) up to 1.8 m/s in the most severe case (patient 8, with an invasive FFR of 0.29), with most patients exhibiting localised jet velocities between 0.8 and 1.1 m/s. In addition, it is possible to see that patients with more severe stenoses, such as patients 1, 8, and 9, exhibit the highest systolic peak velocities, supporting the likelihood of functionally relevant stenoses in these cases. Outside the stenotic regions, the general vessel body remains in the 0.2m/s to 0.5 m/s range across the cohort, confirming that the high-velocity jets are highly localised phenomena driven by the area constriction. This aspect is visually clearer in more severe stenoses, like patients 1, 2, 3, 8, 9, and 10, where the red-coloured jet regions are concentrated and well-defined. In contrast, patients with higher FFR values, such as patients 6 and 11, exhibit smoother and predominantly blue-green distributions. Hence, the visualization of velocity contours is an effective way for the non-invasive and quick assessment of lesion severity and its functional impact on blood flow.

Moreover, since the velocity spatial and temporal behavior changed as expected, with conclusions similar to the ones verified when analyzing the pressure distributions, these findings are aligned with established physiological flow behaviors reported in the literature. Hence, the numerical simulations seem to be accurate: Overall, the velocity fields under hyperemia clearly delineate the influence of coronary geometry on local flow behavior. The velocity behavior of all patients during the systole is physiologically coherent and aligns with coronary dynamics that are expected under maximal vasodilation, supporting the validity of the simulated flow regime used for FFR computation.

### 3.3 Non-invasive fractional flow reserve prediction assessment

The FFR results are displayed in **Table 5**. A comparison of numerical and HeartFlow^®^ FFR values against invasive FFR across all patients was also performed.

**Table 5:** Invasive, numerical and HeartFlow^®^ Fractional Flow Reserve values of all patients.

Patient	1	2	3	4	5	6	7	8	9	10	11	12
Numerical FFR	0.532	0.777	0.589	0.566	0.776	0.921	0.491	0.286	0.801	0.861	0.811	0.738
Invasive FFR	0.58	0.81	0.61	0.6	0.81	0.9	0.5	0.29	0.79	0.9	0.77	0.77
HeartFlow^®^ FFR	0.59	0.78	0.60	0.66	0.79	0.81	0.72	0.65	0.71	0.87	0.97	0.67
Relative error (%) Numerical-Invasive	8.28	4.07	3.44	5.67	4.20	2.33	1.80	1.38	1.39	4.33	5.32	4.16
Relative error (%) HeartFlow^®^-Invasive	1.72	3.70	1.64	10.00	2.47	10.00	44.00	124.14	10.13	3.33	25.97	12.99
Absolute error Numerical-Invasive	0.048	0.033	0.021	0.034	0.034	0.021	0.009	0.004	0.011	0.039	0.041	0.032
Absolute error HeartFlow^®^-Invasive	0.01	0.03	0.01	0.06	0.02	0.09	0.22	0.36	0.08	0.03	0.2	0.1

The relative error was calculated for the numerical and HeartFlow^®^ FFR predictions when compared to the invasive value (considered the gold standard). In the last two rows of the table, where the relative error of the Numerical-Invasive FFRs and HeartFlow^®^-Invasive FFRs is performed, the bold cells correspond to the lowest relative error.

**Figure 5:**
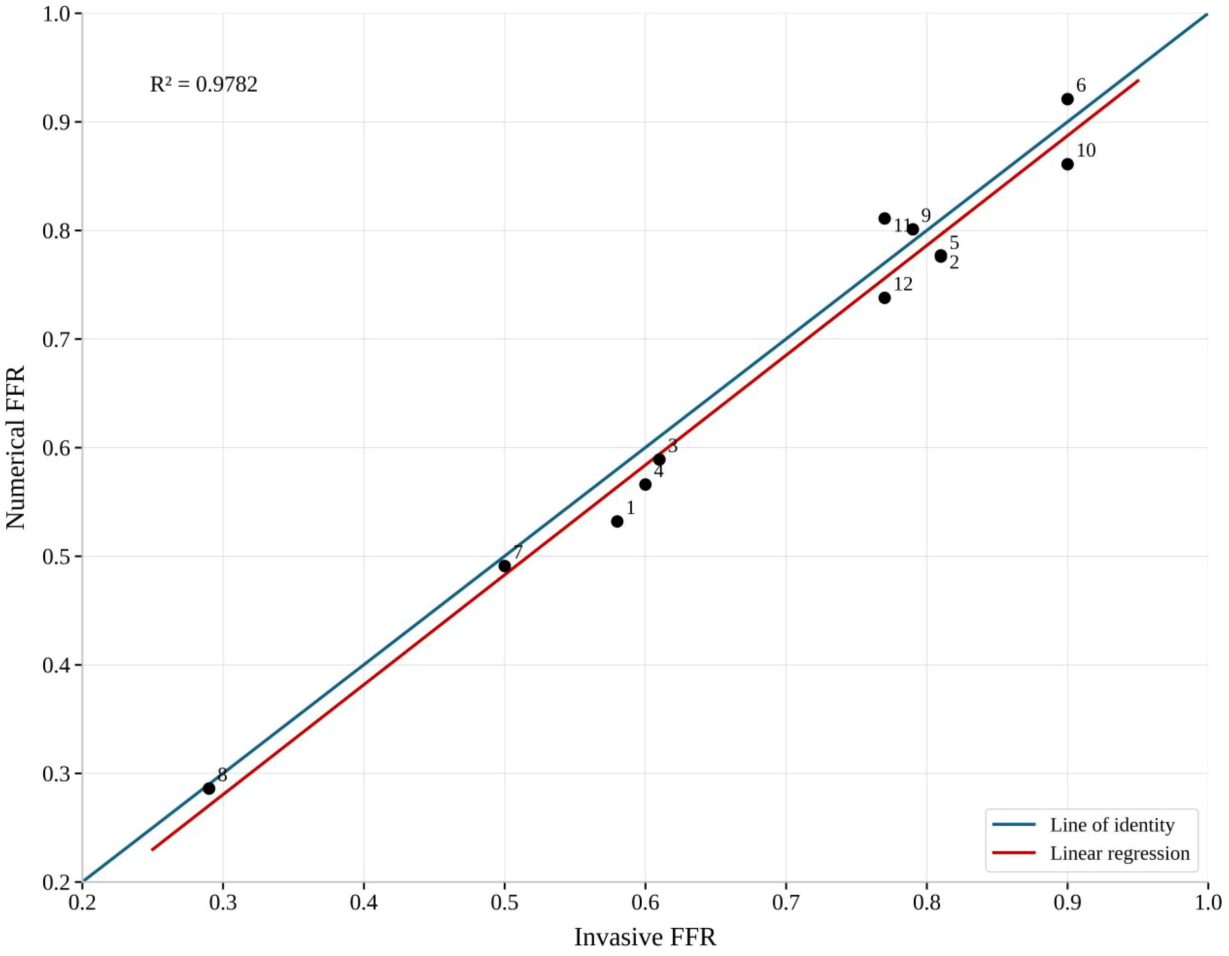
Correlation between numerical FFR and invasive FFR measurements.

Across all 12 patients, the computed non-invasive FFR values seem to demonstrate good alignment with invasive FFR measurements. The relative errors were consistently low, ranging from 1.38% to 8.28%. This level of agreement, within the current limited patient cohort, is encouraging and suggests that the choice of boundary condition models and viscoelastic blood rheological model is appropriate for this type of patient-specific simulation. A correlation analysis is shown in **Figure 5**. R^2^ is equal to 0.9782, indicating a promising linear relationship between numerical and invasive FFR values within this preliminary cohort.

Furthermore, the lowest relative errors were observed in patients 7, 8, and 9, where relative errors fell below 2%. When compared against HeartFlow^®^ and the invasive standard, the developed numerical tool demonstrates higher result stability. Both the developed numerical tool and HeartFlow^®^ generally align with the invasive FFR value, but the developed CFD model achieved a more consistent error profile. Notably, HeartFlow^®^ exhibited substantial fluctuations in accuracy, with errors reaching as high as 44% and 124% in patients 7 and 8, respectively. These can be considered outliers and therefore could play a role in increasing the predictive error of the HeartFlow^®^ results observed for the current patient cohort. In patient 11, HeartFlow^®^ overestimated the FFR (0.97 vs. 0.77 invasive) which would have resulted in a clinically misclassification, failing to identify this functionally severe lesion. The larger discrepancies in the developed numerical model were observed for patient 1 (8.28%) and patient 4 (5.67%). The potential sources for these deviations could be limitations in capturing patient-specific microvascular response and user-dependent segmentation, which may introduce geometric inaccuracies in the reconstructed models.

To provide a more stable measure of error when invasive FFR values are very low, absolute errors are also reported in **Table 5**. The mean absolute error for the numerical model is 0.027 ± 0.014 (range: 0.004-0.048), and for HeartFlow^®^ is 0.101 ± 0.107 (range: 0.010-0.360). The absolute errors for patients 7 and 8 in HeartFlow^®^ are 0.22 and 0.36 respectively, providing a clearer picture of the actual deviation than percentage-based metrics alone.

A binary diagnostic classification at the clinically relevant FFR ≤ 0.80 threshold was performed for both models (numerical and HeartFlow^®^), using invasive FFR as the reference standard. For the numerical model, the sensitivity was 75.0%, specificity 50.0%, accuracy 66.7%, PPV 75.0%, and NPV 50.0%, with 2 false positives (patients 2 and 5, numerical FFR 0.777 and 0.776, respectively, vs. invasive FFR 0.81 for both) and 2 false negatives (patients 9 and 11, numerical FFR 0.801 and 0.811, respectively, vs. invasive FFR 0.790 and 0.770, respectively). For HeartFlow^®^, the sensitivity was 87.5%, specificity 50.0%, accuracy 75.0%, PPV 77.8%, and NPV 66.7%, with the same 2 false positives (patients 2 and 5) and 1 false negative (patient 11, HeartFlow^®^ FFR 0.97 vs. invasive FFR 0.77). Both models share identical false positives in patients 2 and 5, both with invasive FFR of 0.81, just marginally above the 0.8 clinical threshold, suggesting that these misclassifications are due to the FFR value being close to the threshold value, and not necessarily due to large absolute FFR errors. The key difference in performance between the two models lies in patient 9 (invasive FFR equal to 0.79), correctly identified as ischemic by HeartFlow^®^ (HeartFlow^®^ FFR = 0.71) but missed by the numerical model (FFR = 0.801). Patient 11 (invasive FFR equal to 0.77) was missed by both models, though with a substantially larger error by HeartFlow^®^ (HeartFlow^®^ FFR = 0.97). Within this small cohort, both approaches exhibit comparable specificity, while HeartFlow^®^ achieves higher sensitivity, driven by the correct classification of one additional borderline case. It is important to highlight that these differences should be interpreted as preliminary observations given the limited sample size of only 12 patients.

Further analyzing the data of the previous table, it is possible to conclude that the average relative error between numerical and invasive FFR in percentage is 3.86% ± 2.01%, while the average relative error between HeartFlow^®^ and invasive FFR in percentage is 20.84% ± 34.79%. The median average relative error between numerical and invasive FFR is 4.12%, while the median relative error between HeartFlow^®^ and invasive FFR is 10.0%. Furthermore, the maximum error for the numerical model peaked at only 8.28% (Patient 1), whereas HeartFlow^®^ reached errors exceeding 120%. Within this limited patient cohort, the developed numerical tool showed a more consistent error profile than HeartFlow^®^ for the current sample of patients, with lower average error and standard deviation. It must be noted, however, that HeartFlow^®^ operates as a proprietary closed-source commercial platform and does not disclose its specific underlying equations, boundary condition parameters, or meshing protocols. Consequently, a direct comparative analysis of the underlying sources of error is not possible, and the observed discrepancies may result from differences in modeling assumptions rather than superior methodology. These results should therefore be interpreted as preliminary and specific to this cohort, and larger multi-center studies are required before definitive conclusions can be drawn. The absence of algorithmic transparency in commercial tools was one of the motivations for developing our locally executable, transparent CFD-based workflow.

**Figure 6:**
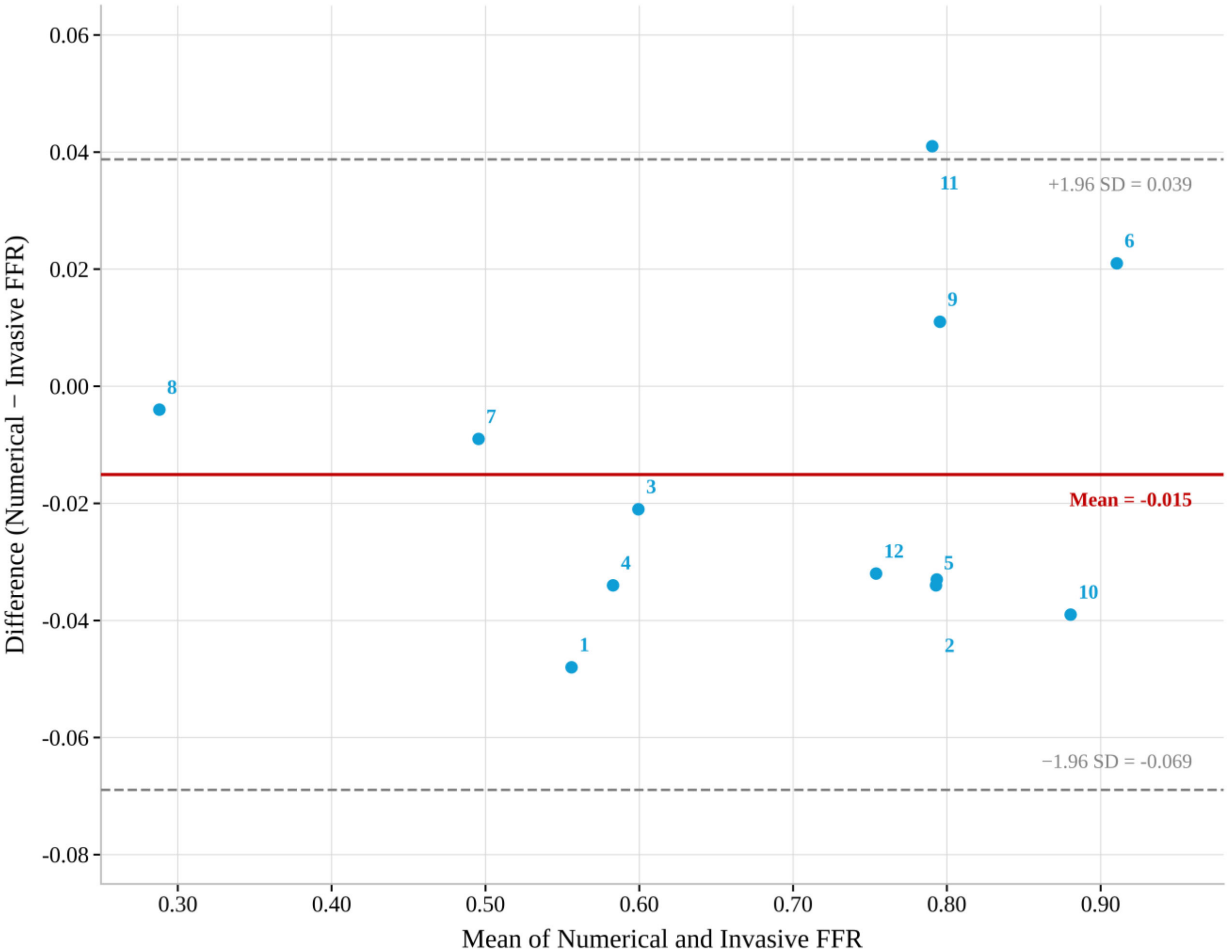
Bland-Altman plot for agreement assessment of numerical and invasive FFR measurements. The red line represents the mean bias (−0.015), and the gray lines are the 95% limits of agreement.

A Bland-Altman analysis (BAA) was performed to assess the degree of agreement between the numerical and the invasive FFR results, as well as identify any systematic bias. BAA shows that this numerical tool has a minimal mean underestimation of −0.015 (95% CI: −0.033 to +0.002), with 95% limits of agreement of −0.069 (95% CI: −0.099 to −0.039) and +0.039 (95% CI: +0.009 to +0.069), suggesting good agreement between the methods. The resulting Bland-Altman plot is shown in **Figure 6**.

It should be noted that Bland-Altman analysis assesses agreement and potential systematic bias. It does not by itself establish diagnostic precision or clinical accuracy.

Overall, this CFD approach shows a tendency to underestimate the FFR when compared to invasive values. This could be a result of the boundary conditions and rheological model being more conservative estimators of flow properties, in this case the pressure, than the invasive measurements. During the invasive method, the pressure wire can affect flow and lead to increased downstream resistance, which could potentially increase the measured invasive FFR. Hence, the slightly lower numerical values could even represent an undisturbed and natural representation of real coronary hemodynamics. However, the underestimations remain within acceptable diagnostic margins and primarily reflect differences in measurement approach rather than fundamental model error.

Although experimental measurements of individual outlet flow splits, as well as inlet and outlet waveforms, were not available for direct validation in this patient dataset, the good agreement between the numerical and invasive FFR values serves as an indirect confirmation of the accuracy of the outlet flow splits, coronary pressure waveforms, and distal hemodynamics. Since the FFR value is inherently impacted by the complex interaction of these quantities, achieving good predictive accuracy in FFR confirms that the boundary conditions and underlying hemodynamic model are operating effectively within the context of this study. Furthermore, the novelty of this work lies in the simultaneous integration of patient-specific Windkessel boundary conditions, a Womersley velocity profile, and the sPTT viscoelastic blood rheology model within a single, end-to-end, computationally efficient framework.

The results of this study suggest that, within this preliminary patient cohort, the developed locally executable and transparent tool has the potential to be a valuable tool for the non-invasive assessment of LCA blood flow and stenoses, supporting clinicians in the diagnosis and management of coronary artery disease. These findings should be regarded as a proof-of-concept validation, and further large-scale studies with more patient cases are needed to confirm the generalizability of these results.

## 4 Conclusions

This study focuses on developing and validating a non-invasive numerical method to estimate Fractional Flow Reserve in patient-specific left coronary arteries. Several patients (twelve) were analyzed to validate the results obtained by the developed computational framework.

Different steps were taken to calculate the FFR from the CT images, which involved segmentation of 3D coronary artery models from CT scans using SimVascular, the programming of user-defined functions of the boundary conditions considering patient-specific properties measured at the hospital, and the incorporation of these features in the numerical simulation software ANSYS^®^. In particular, a Womersley inlet velocity profile and three-element Windkessel model for outlet pressure were used to replicate patient-specific physiological conditions, closer to the real conditions than generic models. To further ensure improved accuracy of the results obtained through the numerical simulations, the simplified Phan-Thien–Tanner (sPTT) viscoelastic model for blood rheology was used to capture the complexity of arterial blood flow.

This study demonstrates that the developed numerical framework represents a promising preliminary proof-of-concept for the non-invasive functional assessment of left coronary arteries with stenoses, within the current limited cohort. The performed numerical simulations successfully captured complex hemodynamic patterns. Furthermore, mapping the velocity and pressure distribution in the systole proved useful for qualitatively assessing the functional severity of stenoses.

When compared with both invasive measurements and HeartFlow^®^ predictions, the proposed numerical framework demonstrated a consistent error profile across the analyzed cohort. While both approaches generally followed similar trends relative to invasive FFR values, HeartFlow^®^ showed greater variability in accuracy across individual cases. In particular, larger deviations were observed in specific patients, highlighting potential variability in performance depending on anatomical or physiological factors.

The numerical FFR predictions showed strong agreement with invasive measurements, with a low average relative error and high correlation. Within the analyzed cohort, the proposed framework demonstrated improved consistency compared to HeartFlow^®^, which exhibited greater variability in relative error.

These results highlight the potential of the proposed approach as a cost-effective and transparent computational alternative for non-invasive FFR estimation. However, further large-scale validation studies are required before clinical translation.

By integrating physiologically informed boundary conditions close to reality along a viscoelastic rheological model, the developed tool demonstrated encouraging predictive performance within this preliminary cohort. These findings highlight the potential of the developed locally executable, transparent numerical tool as a cost-effective non-invasive alternative for FFR estimation. The current study should be understood as a successful preliminary proof-of-concept validation, and further large-scale, multi-center clinical trials are required to fully establish the accuracy, robustness, and clinical readiness of this framework.

## 5 Study limitations

Although the current methodology was validated across a cohort of patient-specific left coronary arteries, increasing the number of patients in the dataset is essential to improve the statistical validation that is required for clinical implementation of this tool. The current patient cohort is too small to effectively state that it is more accurate than existing FFR calculation methods, and many more patients should be tested. Therefore, a larger multi-center study, involving collaborations across multiple hospitals, would be necessary to ensure the generalizability and accuracy of the model.

Regarding 3D model creation, it is known that the quality of the final 3D model is inherently impacted by the resolution of the available CT scans. Some CT images had some noise, which can occur in medical imaging due to patient movement, respiratory motion, low radiation dose protocols, dense calcifications, among others. These result in small vessels and highly calcified regions (which cause blooming artifacts in the images) difficult to segment accurately, altering local pressure gradients which can affect the final FFR value.

While the current pipeline focuses primarily on validating the core simulation methodology, further optimization would enhance its practicality for hospital settings. In this study, the reconstruction of geometries was performed through manual image segmentation, which requires significant user dependency and requires approximately 48 hours per case. Hence, the integration of automated reconstruction and deep learning (DL) methods could lead to the reduction to minutes or even seconds without affecting the quality of the output vessel 3D geometry. However, to ensure their clinical viability, the implementation of these algorithms must incorporate explainability and transparency, which are challenges present in existing commercial tools. It is important to highlight that the obtained numerical results are compared to established tools like HeartFlow^®^ based solely on the final diagnostic accuracy of the FFR values, rather than a direct comparison of the end-to-end processing time from initial CT acquisition.

The implementation of artificial intelligence in FFR prediction is the next step of the authors, and the approach is mentioned in the Future Works section.

While the current approach is based in established vascular scaling laws and has demonstrated sufficient accuracy for the current patient cohort, the outlet Windkessel modelling could be improved. The reduced number of elements of the used lumped-parameter model could lead to an overly simplification of coronary circulation. Therefore, other complex models with more elements should be explored.

The hyperemia modeling strategy applies uniform physiological scaling factors to all patients, following the established methodology of (Wilson et al., 1990). However, this represents an approximation, as potential patient-to-patient variability in microvascular dilation response could introduce discrepancies between the computational 3D models and the real coronary anatomy in individual cases.

Furthermore, the manual adjustments made to avoid intersections between vessels after the uniform cross-sectional area scaling introduce a geometric approximation whose potential impact on local pressure gradients and FFR values cannot be fully quantified without dedicated sensitivity analyses. The low FFR prediction errors observed across the cohort suggest that this approximation does not introduce systematic bias, but this remains a limitation that should be addressed in future work.

Manual segmentation, manual smoothing, and manual distal plane selection introduce substantial operator dependency. The lack of reproducibility or interobserver variability analysis is an important limitation of the current workflow. Incorporating semi-automated or automated segmentation techniques will be a crucial step in future iterations to minimize user bias and ensure the scalability of the tool in standard clinical settings; this is currently being developed by our research group.

The current study did not quantify the individual contribution of each model component (Windkessel boundary conditions, Womersley inlet, and sPTT viscoelastic rheology) to predictive performance. Quantifying these contributions was not the objective of this study, as the goal was to validate the complete integrated FFR calculating framework. However, future sensitivity analyses would further strengthen the methodological justification of each component.

## 6 Future works

Future research will focus on transitioning the current computational framework into an automated clinical diagnostic of atherosclerosis by the prediction of the FFR. As seen before, this would eliminate the time and manual constraints of the current solution.

Aiming to reduce the time-consuming and manual nature of segmenting and obtaining the 3D geometries, the authors are currently developing a semi-automated approach in Python that automatically finds the different branches, their centerline, the cross-sectional area at several centerline points, as well as the automatic differentiation between the inlet and outlet surfaces. Furthermore, using the previous information, the definition of hyperemic conditions can also be established in an automated way. The implementation of the semi-automated approach could eventually eliminate the manual reconstruction stage, the absence of reproducibility or interobserver variability analysis, and reduce the process of obtaining geometries to a few minutes requiring only minimal intervention. The usage of this tool instead of the manual image segmentation process would eliminate most of the current disadvantages, including the need to perform a dedicated sensitivity analysis on the impact of the hyperemia scaling procedure on computed FFR values, as the process would not be manual.

The next step is the development of a DL algorithm that uses geometry information obtained in the Python method with clinical information provided by the medical doctors to return as output a prediction of the FFR value and replace the computationally intensive CFD simulations.

The objective of the present study is to develop a tool that predicts the FFR in reasonable time enabling its usefulness in real clinical scenarios. More patient cases need to be added to the current sample, by collecting from other hospitals and regions to increase generalizability. The data set is to be divided into a training, validation and testing set to ensure a good performance of the model.

Using AI would eliminate the need to time-consuming and expensive computational hardware in hospitals and software licenses, possibly helping the diagnosis and improving patient outcomes.

## Data Availability

The original contributions presented in the study are included in the article/supplementary material. Further inquiries can be directed to the corresponding author.
